# Adult Acute Respiratory Distress Syndrome (ARDS) Caused by Human Rhinovirus During Janus Kinase Inhibitor Therapy for Rheumatoid Arthritis: A Case Report and Literature Review

**DOI:** 10.7759/cureus.94213

**Published:** 2025-10-09

**Authors:** Bodhisatwa Choudhuri, Simontini Patra, Pratik Biswas, Anindya Dasgupta, Nishant Agarwal, Sujoy Das Thakur

**Affiliations:** 1 Critical Care and Rheumatology, Parkview Super Specialty Hospital, Kolkata, IND; 2 Laboratory Medicine, Employees' State Insurance Post Graduate Institute of Medical Sciences and Research (ESI-PGIMSR), Kolkata, IND; 3 Pulmonology, ILS Hospitals, Howrah, IND; 4 Emergency Medicine, Narayana Hospital Barasat, Barasat, IND; 5 Emergency Medicine, Charnock Hospital, Kolkata, IND; 6 Emergency Medicine, Manipal Hospitals, Kolkata, IND

**Keywords:** adult respiratory distress syndrome, atypical pneumonia, awake prone position, case report, janus kinase inhibitors, literature review, pneumonia, rheumatoid arthritis, rhinovirus, tofacitinib

## Abstract

An adult with seropositive rheumatoid arthritis (RA) receiving methotrexate (MTX) and tofacitinib, a Janus kinase (JAK) inhibitor, developed rapidly progressive hypoxemic respiratory failure following a brief coryzal prodrome. High-resolution CT showed diffuse bilateral ground-glass opacities with dependent consolidation. An upper-airway syndromic multiplex PCR detected human rhinovirus (HRV)/*Enterovirus*, while other pathogens were excluded. The clinical tempo, virologic confirmation, and imaging pattern favored viral acute respiratory distress syndrome (ARDS); drug-related pneumonitis and RA-associated interstitial lung disease remained key differentials. Management included temporary withdrawal of disease-modifying therapy, high-flow nasal oxygen with prolonged awake proning, intermittent non-invasive ventilation during episodes of worsening dyspnoea, a conservative fluid strategy, early de-escalation of empiric antibiotics when cultures remained negative, and a short course of systemic corticosteroids. The patient improved without intubation, was weaned from oxygen, and was discharged in stable condition. MTX was reintroduced without pulmonary relapse; leflunomide was added for residual articular activity. After shared decision-making and due to the patient's aversion to injectables, tofacitinib was restarted, resulting in continued respiratory stability and radiographic resolution on follow-up. This case underscores practical diagnostic discriminators and a stepwise approach to temporarily withholding and safely reintroducing immunosuppression in HRV-ARDS complicating RA treatment.

## Introduction

Human rhinovirus (HRV) is a single-stranded RNA picornavirus belonging to the genus *Enterovirus* (EV). It is increasingly recognized as a clinically significant cause of lower respiratory disease in adults, including severe pneumonia and intensive care unit (ICU) admission. In population-based surveillance, HRV accounted for ~11% of adult acute respiratory illnesses and ~3 hospitalizations per 1,000 adults annually (second only to influenza) [1-5]. By contrast, adult HRV-associated acute respiratory distress syndrome (ARDS) remains rare and is reported almost exclusively as isolated case reports or small series; robust population-level incidence estimates are lacking [1-9]. The risk is amplified by host factors such as immunosuppression and impaired type I/III interferon signaling. Janus kinase (JAK) inhibition attenuates those antiviral pathways and carries explicit labeling to interrupt therapy during severe infection; yet, the same class improves outcomes in hyperinflammatory COVID-19 pneumonia, illustrating a context-dependent "double-edged sword" [10-13]. Rapid syndromic polymerase chain reaction (PCR), such as the BioFire Respiratory Panel (BioFire Diagnostics, LLC, Salt Lake City, UT, USA), can detect HRV/EV within hours from an upper-airway specimen; however, the combined analyte and qualitative readout require clinico-radiologic correlation before causality is inferred [14].

## Case presentation

A 44-year-old woman with seropositive rheumatoid arthritis (RA) (rheumatoid factor and anti-cyclic citrullinated peptide strongly positive) had very high baseline disease activity (DAS28-CRP 6.3) [15,16]. She was obese (body-mass index ≈41), a non-smoker, and a non-alcoholic. Comorbidities were hypothyroidism, hypertension, and dyslipidaemia. Vaccinations (influenza, pneumococcal, and COVID-19) were up to date. Long-term therapy comprised MTX 10 mg orally once weekly for 18 months with folic acid 5 mg twice weekly and tofacitinib 5 mg twice daily for six months without dose changes. There had been no prior MTX toxicity and no chronic glucocorticoid or biologic use. Her other regular home medications included oral atorvastatin 10 mg once daily, telmisartan 40 mg once daily, and levothyroxine 75 mg once daily.

**Figure 1:**
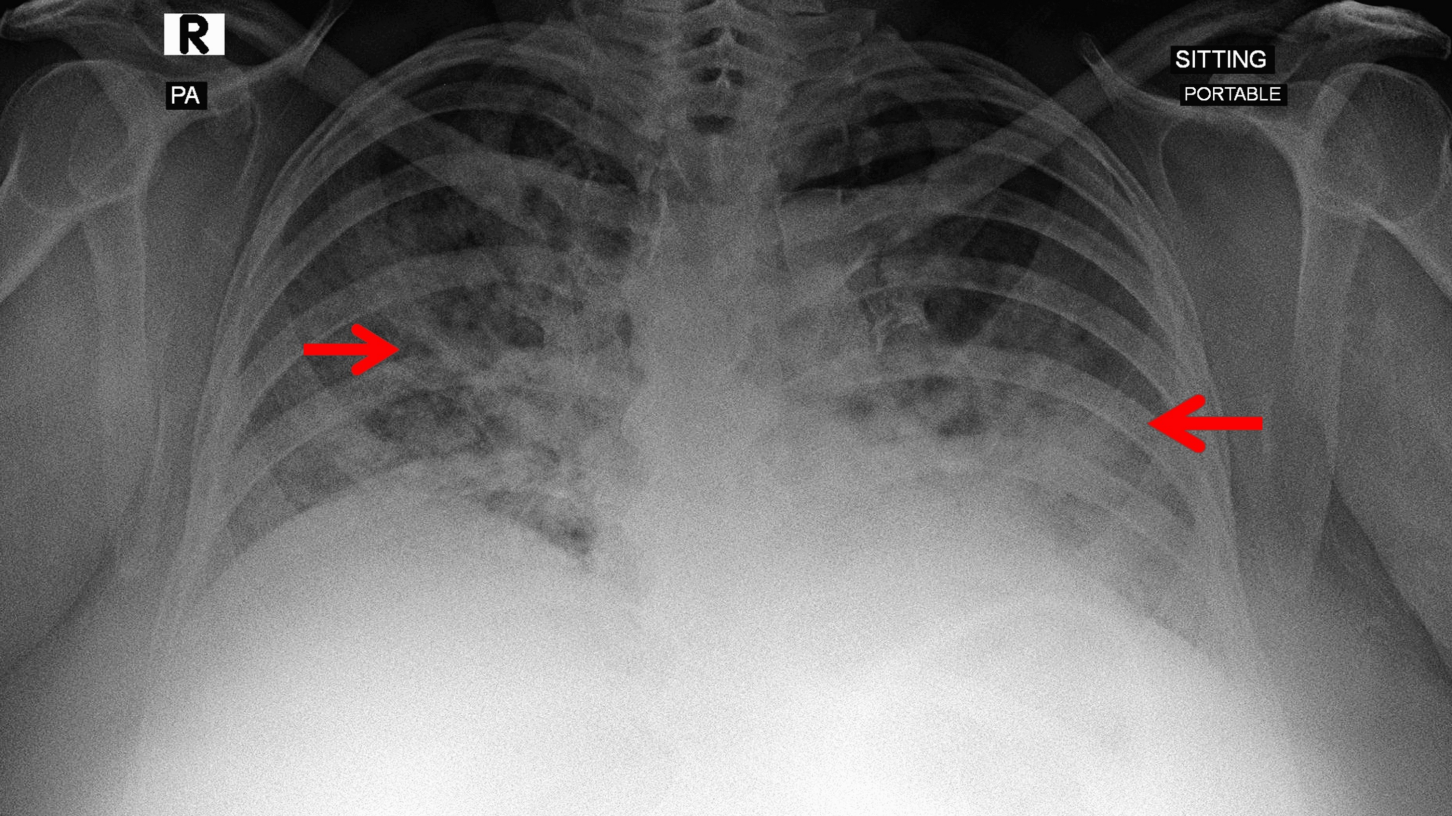
Admission chest radiograph anteroposterior (portable) view The initial chest radiograph at presentation shows patchy, bilateral airspace opacities with lower-zone predominance, consistent with acute diffuse lung involvement. Red arrows indicate representative bilateral patchy opacities.

**Figure 2:**
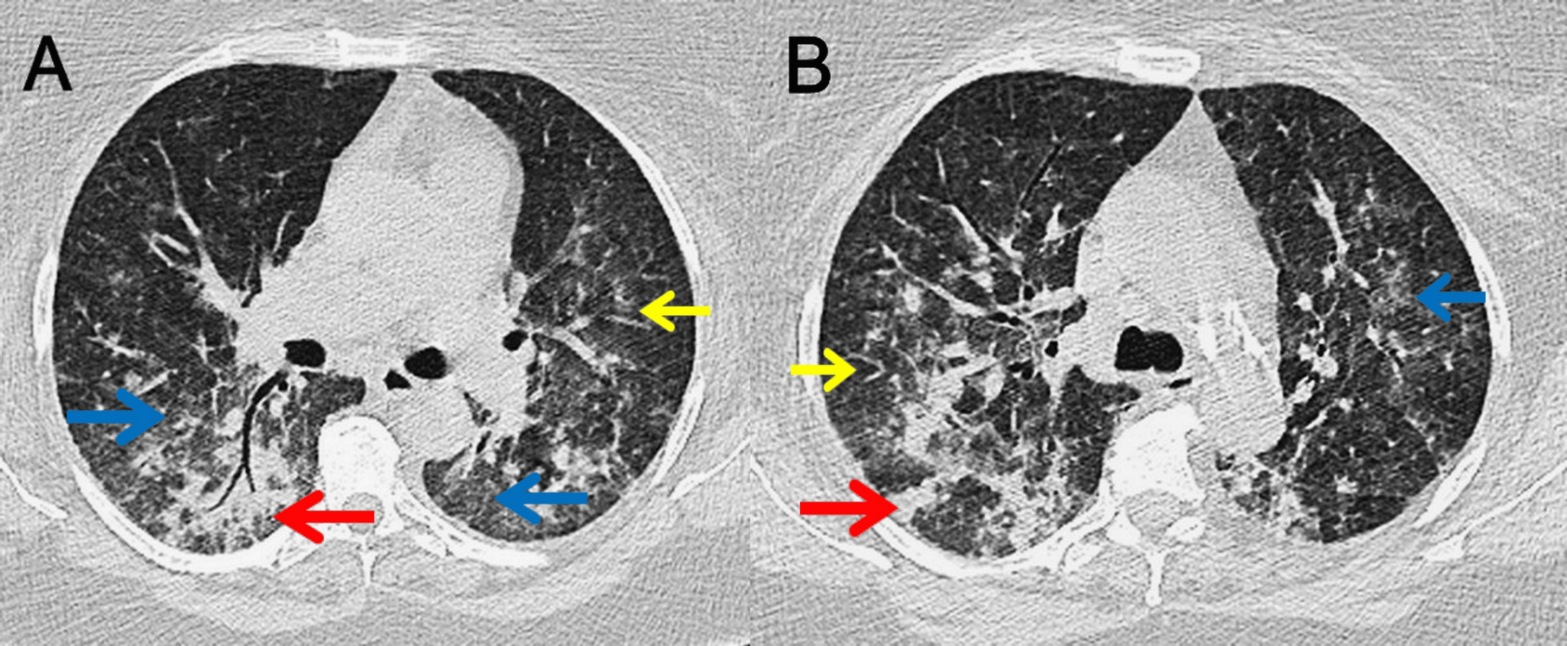
HRCT of the thorax on Day 1 A (basal): Lower-lobe predominant dependent posterior consolidation on diffuse bilateral GGO with mild interlobular septal thickening; no pleural effusion or traction bronchiectasis. B (mid-thoracic): Patchy peripheral/peribronchovascular GGO with subtle reticulation; less consolidation than the bases. The arrows identify key abnormalities: red highlights areas of consolidation, blue marks GGO, and yellow indicates interlobular septal thickening. Overall pattern, in context, supports ARDS. GGO: ground-glass opacities, ARDS: acute respiratory distress syndrome

Five days before admission, she developed coryzal symptoms and a dry cough, followed by fever and rapidly progressive dyspnoea over 48 hours. There were no similar symptoms among household members or close contacts in the preceding weeks. The patient did not work in a child-facing environment (i.e., no school/day-care or pediatric healthcare exposure). In the emergency department, she was febrile, tachypneic with accessory-muscle use (respiratory rate ~32/min), hypoxemic on room air (peripheral oxygen saturation 86%), tachycardic (~114/min), and normotensive (132/78 mmHg). Chest radiography showed new bilateral air-space opacities (Figure 1). Admission laboratory results and arterial blood gas indices are summarised in Table 1. In brief, there was neutrophilic leukocytosis with markedly elevated inflammatory markers, a low-intermediate procalcitonin level, and a mild, transient rise in serum creatinine, consistent with early acute kidney injury. High-resolution CT (HRCT) of the thorax on Day 1 demonstrated diffuse bilateral ground-glass opacities (GGO) with mild interlobular septal thickening and confluent dependent posterior lower-lobe consolidations, without pleural effusion or traction bronchiectasis, an appearance compatible with diffuse alveolar damage in the acute phase (Figure 2).

**Table 1:** Serial laboratory parameters and ABG indices from admission (Day 0) to discharge (Day 12) in HRV-associated ARDS Daily laboratory values and arterial blood gas indices from Day 0–12; ABGs reflect contemporaneous respiratory support, PaO₂/FiO₂ calculated from recorded FiO₂, reference ranges in parentheses, and “—” denotes not measured. ESR: erythrocyte sedimentation rate, LDH: lactate dehydrogenase, IL-6: interleukin-6, FEU: fibrinogen-equivalent units, INR: international normalized ratio, AST: aspartate aminotransferase, ALT: alanine aminotransferase, FiO₂: fraction of inspired oxygen, PaCO₂: partial pressure of arterial carbon dioxide, PaO₂: partial pressure of arterial oxygen, NT-proBNP: N-terminal pro-B-type natriuretic peptide, hs-Troponin I: high-sensitivity troponin I

Parameters	Day 0 (admission)	Day 3	Day 8	Day 12 (discharge)	Normal range
Haemoglobin (g/dL)	13.5	12.4	12.8	13.1	12–16
Total leucocyte count (×10^9/L)	11.6	12.4	8.7	7.1	4.0–11.0
Neutrophils (%)	78	84	72	62	40–80
Lymphocytes (%)	12	8	20	28	20–40
Platelets (×10^9/L)	170	145	220	280	150–400
C-reactive protein (mg/L)	164	211	63	12.8	<5
Procalcitonin (ng/mL)	0.17	-	0.06	-	<0.10
ESR (mm/h)	48	60	38	22	<20
Ferritin (ng/mL)	950	1,100	480	-	30–400
LDH (U/L)	420	-	320	-	140–280
IL-6 (pg/mL)	-	55	-	6	<7
D-dimer (mg/L FEU)	1.8	2.3	-	0.4	<0.5
Fibrinogen (mg/dL)	580	620	450	380	200–400
INR	1.1	1.2	1.1	1	0.8–1.2
Urea (mg/dL)	56	67	32	28	15–45
Creatinine (mg/dL)	1.3	1.1	0.9	0.8	0.6–1.3
Sodium (mmol/L)	134	132	136	138	135–145
Potassium (mmol/L)	4.7	5.1	4.2	4	3.5–5.0
Bicarbonate (mmol/L)	18	20	24	25	22–28
AST (U/L)	60	72	45	35	<40
ALT (U/L)	48	56	40	32	<41
Total bilirubin (mg/dL)	0.9	1.2	0.8	0.7	0.2–1.2
Albumin (g/dL)	3	2.6	3.2	3.8	3.5–5.0
FiO₂ at time of ABG (fraction)	0.6	0.7	0.3	0.21	-
pH (arterial)	7.48	7.46	7.42	7.4	7.35–7.45
PaCO₂ (mmHg)	25	29	38	42	35–45
PaO₂ (mmHg)	74	54	88	96	80–100*
Lactate (mmol/L)	2.2	2.6	1.6	1.2	0.5–2.0
PaO₂/FiO₂ ratio	123	77	293	457	>300
NT-proBNP (pg/mL)	220	-	-	-	<125 (<75y)
hs-Troponin I (ng/L)	12	-	-	-	<34

A syndromic multiplex PCR (BioFire Respiratory Panel) from a nasopharyngeal sample detected HRV/EV [14]. SARS-CoV-2 testing was negative on two occasions. Bacterial cultures from blood and sputum and fungal biomarkers (serum (1→3)-β-D-glucan and *Pneumocystis jirovecii* PCR) were negative. Bronchoscopy was deferred because the virologic result aligned with the imaging pattern and the clinical course. She met the Berlin criteria [17] for moderate ARDS, based on an acute onset within one week of a clear insult, bilateral infiltrates not explained by cardiac failure, and a PaO₂/FiO₂ ratio of 100-200 on high-flow support. Table 2 summarises a day-by-day clinical course that includes diagnostics, respiratory support, treatment modifications, and follow-up.

**Table 2:** Clinical timeline from prodrome to six-month follow-up Days are referenced to the index ED/ICU presentation (Day 0). The timeline summarizes key diagnostics, treatment milestones, respiratory support, transitions of care, and outcomes. "→" indicates onward days. ARDS: acute respiratory distress syndrome, CXR: chest radiograph, DMARD: disease-modifying antirheumatic drug, ED: emergency department, EV: *Enterovirus, *HFNC: high-flow nasal cannula, HRCT: high-resolution computed tomography, HRV: human rhinovirus, ICU: intensive care unit, JAK: Janus kinase, LFN: leflunomide, MTX: methotrexate, NIV: non-invasive ventilation, PCR: polymerase chain reaction, RA: rheumatoid arthritis, RA-ILD: rheumatoid-arthritis-associated interstitial lung disease, VTE: venous thromboembolism

Day	Event	Details (diagnostics, interventions, outcomes)
−3 to −1	Prodrome	Coryzal symptoms with low-grade fever and progressive dyspnea; self-care at home.
0	ED/ICU presentation	Acute hypoxemic respiratory failure meeting Berlin ARDS criteria. HRCT Day 1: diffuse bilateral ground-glass opacities with dependent posterior consolidations (Figure 2).
0	Initial management	JAK inhibitor held. Supportive care with HFNC and awake proning (brief NIV as needed). Conservative fluids, VTE prophylaxis, and empiric antibiotics pending microbiology.
1–2	Microbiology confirmation	Upper-airway multiplex PCR positive for HRV/EV; other viral/bacterial/atypical panels negative.
2	Steroid initiation	IV corticosteroid course started based on ARDS trajectory and inflammatory phenotype.
3 →	Clinical improvement	Improving oxygenation and work of breathing from Day 3 onward. No intubation required.
1–7	ICU course	HFNC with awake proning; pulmonary physiotherapy. Gradual reduction in oxygen requirement.
8	Ward shift	Transferred from ICU to ward, steroids tapered to oral, antibiotics de-escalated
8–12	Ward course	Mobilization, incentive spirometry, and weaning oxygen.
10	Off oxygen	Maintained oxygen saturation on room air during activity and rest.
12	Discharge	Clinically stable on room air; safety-netting. Plan for the reintroduction of DMARDs under rheumatology supervision.
+7 days	Outpatient follow-up	No relapse; MTX restarted.
6 weeks	Rheumatology review	No relapse but inadequate RA control; LFN added to MTX.
4 months	Rheumatology review	Persistent suboptimal RA control; tofacitinib added with MTX; LFN stopped.
6 months	Rheumatology review	Stable and asymptomatic; ongoing monitoring.

The patient was admitted to the ICU. Tofacitinib and MTX were withheld on arrival. Respiratory support began with high-flow nasal cannula (HFNC) at 50-60 L/min, with a fraction of inspired oxygen (FiO₂) of 0.6-0.8, combined with prolonged awake proning (≥8 hours/day) and conservative fluid management. During episodic desaturation or work-of-breathing surges, non-invasive ventilation (NIV) was applied intermittently (short sessions with low tidal-volume targets and cautious positive end-expiratory pressure), after which she was stepped back to HFNC. Vasopressors and endotracheal intubation were not required.

Empirical piperacillin-tazobactam plus clarithromycin was started for severe community-acquired pneumonia risk and de-escalated once cultures and biomarkers remained negative. Systemic corticosteroids were administered as intravenous methylprednisolone 125 mg daily for three days, then 40 mg twice daily for three days, followed by an oral taper as oxygenation improved. Deep venous thrombosis (DVT) and stress ulcer prophylaxis, incentive spirometry, and early mobilization were instituted according to unit protocol.

Pulmonary embolism (PE) was considered; however, the pre-test probability was low (no haemoptysis, no clinical signs of DVT, and an alternative diagnosis was more likely), and the D-dimer was only mildly elevated, which is a common finding in acute infections. The patient’s hypoxaemia correlated with diffuse bilateral parenchymal involvement on imaging, and focused echocardiography showed no right-ventricular strain. In view of the low probability of PE, the limited specificity of a mildly elevated D-dimer, and the risks associated with transport in early ARDS, CT pulmonary angiography was not pursued. Standard DVT prophylaxis was maintained with ongoing clinical surveillance.

**Figure 3:**
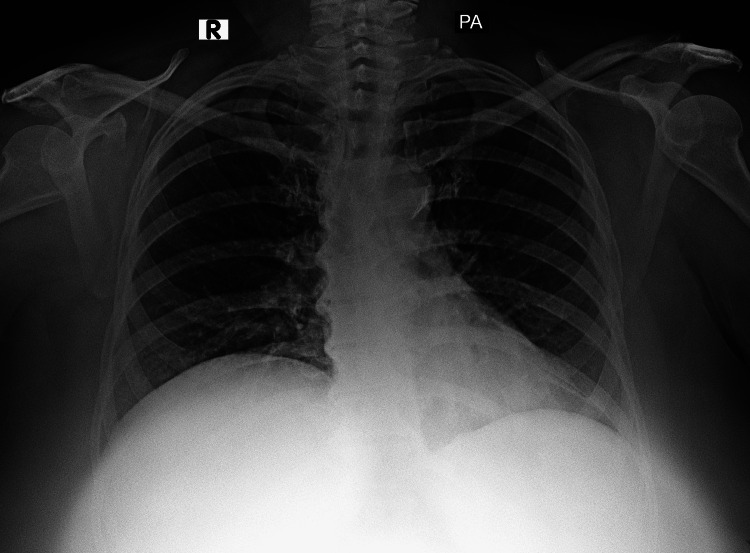
Follow-up chest radiograph posteroanterior view showing radiographic resolution Chest radiograph at six-month follow-up demonstrates clear lung fields with resolution of the prior bilateral air-space opacities; no pleural effusion or pneumothorax is seen, and the cardiomediastinal silhouette is within normal limits.

Hypoxaemia and dyspnoea improved steadily; by Day 10, she no longer required supplemental oxygen. Intensive care and hospital stays were eight and twelve days, respectively. She was discharged on tapering steroids and five days of oral cefuroxime 500 mg twice daily. MTX 10 mg weekly was restarted a week after discharge without pulmonary relapse; leflunomide 20 mg was added at six weeks for residual articular activity. As she continued to be symptomatic, tofacitinib was restarted after four months following detailed counselling, as she declined injectable biologic agents. At six months, she was asymptomatic with normal resting oximetry and interval radiographic resolution (Figure 3).

## Discussion

Review of relevant literature

We performed a focused search of PubMed/MEDLINE, Google Scholar, and Scopus for adult reports of HRV-associated ARDS (HRV-ARDS). The search string was (“human rhinovirus” OR “rhinovirus” OR “enterovirus”) AND (“acute respiratory distress syndrome” OR “ARDS”) AND (“adult” OR “adults”), with limits to English, age ≥18 years, and publication years 2010-2025. Pediatric studies were excluded, and the reference lists of included articles were manually screened. Adult HRV-ARDS remains a rare condition, with fewer than 10 published cases [1-9]. Most reports describe middle-aged to older adults [1-3,6,9]; several small series suggest a female tilt [1-2,5,7-9], but numbers are limited. Immunocompromise recurs as a theme, with solid-organ transplantation, chronic lung disease, and iatrogenic immunosuppression being frequent backdrops [2,4-9]. Key case-level characteristics from adult literature are summarized in Table 3.

**Table 3:** Published case reports of HRV-induced ARDS in adults Summary of published adult case reports of HRV-associated ARDS, detailing host factors, diagnostics and specimens, disease severity, respiratory support, adjunct therapies, and outcomes [1-9]. ARDS severity is categorized by the PaO₂/FiO₂ ratio, as per the Berlin definition [17]. APRV: airway pressure release ventilation, ARDS: acute respiratory distress syndrome, BAL: bronchoalveolar lavage, COPD: chronic obstructive pulmonary disease, HFpEF: heart failure with preserved ejection fraction, HFNC: high-flow nasal cannula, HRV: human rhinovirus, LOS: length of stay, LPV: lung-protective ventilation, MTX: methotrexate, MV: mechanical ventilation, NIV: non-invasive ventilation, OSA: obstructive sleep apnea, PCR: polymerase chain reaction, P/F: PaO₂/FiO₂ ratio, RA: rheumatoid arthritis, RP2.1: BioFire Respiratory Panel 2.1, RT-PCR: reverse-transcription PCR

Author/year	Age/gender	Underlying conditions	Clinical setting	Presenting symptoms	ARDS severity (P/F ratio)	Diagnostic method	Respiratory support	Corticosteroids	Hospital stay (days)	Outcome
Soni et al. [7], 2017	22F	Asthma, Turner syndrome	Community-acquired	Respiratory distress	Moderate-Severe	Multiplex PCR	MV + tracheostomy	Yes	45	Recovery with prolonged weaning
Yousefi et al. [4], 2019	35M	Liver transplant recipient	Community-acquired	Fever, cough, dyspnoea	Severe	PCR (nasopharyngeal + BAL)	MV	Yes	28	Recovery
Ayala et al. [9], 2019	60F	COPD, morbid obesity, OSA, HFpEF	Postoperative (nephrolithotomy)	Post-op fever, respiratory distress	Moderate-Severe (P/F 110)	GenMark eSensor respiratory panel	LPV à APRV, MV	Not specified	13	Recovery
Ngu et al. [1], 2019	59F	Immunocompetent	Community-acquired	Rapid respiratory deterioration	Severe	Multiplex PCR	HFNC	Yes	14	Recovery
Revell et al. [8], 2021	25F	Pregnancy (28 weeks)	Community-acquired	Cough, dyspnoea, bronchiolitis	Severe	Multiplex PCR	MV	Yes	Not specified	Recovery
Cecchini et al. [6], 2022	67M	COPD	Community-acquired	Fever, dyspnoea	Moderate	Multiplex PCR	HFNC	Yes	Not reported	Recovery
Hamid et al. [2], 2022	70F	RA on MTX	Community-acquired	Fever, dyspnoea, hypoxemia	Severe (P/F 79)	Multiplex PCR	MV	Yes	Not reported	Recovery
Phan et al. [5], 2023	33F	Liver transplant, Crohn's disease, asthma	Community-acquired	Sore throat, cough, dyspnoea	Severe (P/F 79)	Multiplex PCR	MV	No	5	Complete recovery
Avgoustou et al. [3], 2023	89M	Elderly, afebrile presentation	Community-acquired	Dyspnoea, rapid deterioration	Severe	Multiplex PCR	MV	Yes	Not reported	Death
Current case, 2025	44F	RA on MTX + tofacitinib	Community-acquired	Fever, dry cough, hypoxemia	Severe (P/F ~77)	BioFire RP2.1 PCR	HFNC + NIV	Yes (methylprednisolone)	12	Complete recovery

Beyond individual cases, adult epidemiology and syndromic patterns are summarized in Table 4; cohort data place HRV firmly among serious adult respiratory pathogens while reminding us that progression to frank ARDS is uncommon [18-20]. Advanced age (≥60 years in 90% of severe cases), multiple comorbidities (93.8% of hospitalized patients), and immunosuppression are key risk factors [18]. In a Chilean hospital cohort, HRV accounted for 23.7% of severe acute respiratory infections, most often presenting as community-acquired pneumonia, with ARDS occurring in a small minority [18]. Among hematopoietic-cell transplant recipients, lower-respiratory HRV infection carried a 90-day mortality of 41%, with worse outcomes linked to low monocyte counts, oxygen need at diagnosis, and corticosteroid doses >1 mg/kg/day [19]. Severity in immunocompromised adults has paralleled 2009 H1N1 influenza in some settings, with hospitalization rates near 40% among HRV-positive patients [20]. These patterns, together with the postoperative HRV-ARDS described by Ayala et al. [9], underline that both community-acquired and healthcare-associated acquisitions are relevant. In the Chilean cohort, CURB-65 scores ≥3 were associated with higher mortality (OR 23.4; AUC 0.799), supporting simple bedside risk stratification [18,21].

**Table 4:** Epidemiological studies of HRV respiratory infections in adults Adult epidemiological studies of HRV respiratory infection summarising design, population, setting, detection method, clinical presentations, severe disease/ARDS, mortality, and key findings. Percentages are within-study [18-20]. CURB-65 is calculated per Lim et al. [21] ARDS: acute respiratory distress syndrome, CAP: community-acquired pneumonia, COPD: chronic obstructive pulmonary disease, CURB-65: confusion, urea, respiratory rate, blood pressure, age ≥65 score, H1N1: influenza A/H1N1, HRV: human rhinovirus, ICU: intensive care unit, LRTI: lower respiratory tract infection, PCR: polymerase chain reaction, RT-PCR: reverse-transcription PCR, SARI: severe acute respiratory infection, US: United States, O₂: supplemental oxygen

Study/year	Study design	Population	Sample size	Age (years)	Setting	HRV detection method	Clinical presentations	Severe disease/ARDS	Mortality rate	Key findings
Seo et al. [19], 2017	Retrospective cohort	Hematopoietic cell transplant recipients	697 patients (166 with HRV LRTI)	Adult transplant recipients	US transplant centres	PCR from respiratory specimens	Lower respiratory tract infection	HRV LRTI in 166 patients	90-day mortality: 41%	HRV LRTI mortality comparable to other respiratory viruses; risk factors: low monocytes, O₂‚ requirement, high-dose steroids
Fica et al. [18], 2015	Prospective surveillance	Hospitalized adults with SARI	32 HRV cases (of 135 total viral)	Mean 79.5 (range 49-95)	Chilean hospital	RT-PCR	CAP (68.8%), COPD exacerbation (21.9%), heart failure (6.3%)	Respiratory failure in 78.6%; ICU admission 31.2%	12.50%	HRV ranked 2nd after influenza (23.7% vs 37.8%); CURB-65 ≥3 predicted mortality
Kraft et al. [20], 2012	Retrospective cohort	Immunocompromised adults	35 HRV patients vs 35 H1N1 patients	Not specified	US academic centre	PCR	Respiratory symptoms requiring hospitalization	Hospital admission: 40% HRV vs 37% H1N1	28.6% HRV vs 28.6% H1N1	HRV severity comparable to pandemic H1N1 in immunocompromised

Management across reports is mainly supportive and adheres to standard ARDS protocols. Lung-protective ventilation is the rule [5,6,9]; high-flow nasal oxygen and non-invasive strategies are common where feasible [1,6,8], and airway pressure release ventilation has rescued refractory hypoxemia with successful extubation in individual cases [9]. Corticosteroids were frequently used, albeit without HRV-specific evidence [1-3,5-9]. Mortality across reported series ranges from 12.5% to 41% depending on population risk [3,7,18,19], while survivors typically recover fully, occasionally after protracted weaning [3]. The small literature likely reflects both true rarity and earlier under-recognition before widespread multiplex PCR testing [5,22].

Epidemiology of HRV-associated ARDS in adults

This case adds to the small adult literature showing that HRV, often dismissed as an upper-airway pathogen, can drive fulminant lower-respiratory disease and ARDS. Contemporary adult cohorts of hospitalized non-influenza respiratory viral infections frequently identify HRV, with outcomes similar to influenza, yet frank ARDS remains uncommon and largely confined to case reports [1-9]. Cohort-level observations likewise suggest HRV is common among severe admissions, while progression to ARDS is unusual [1-9,12-14].

JAK inhibition and associated respiratory disease

Host immunobiology likely amplified risk here. JAK-signal transducer and activator of transcription (JAK-STAT) signalling underpins interferon-mediated antiviral defense; pharmacologic JAK inhibition can blunt these pathways and raise susceptibility to serious infection, hence the tofacitinib recommendation to interrupt treatment during active serious infection [10]. Paradoxically, the same agent reduced death or respiratory failure in randomized hospitalized COVID-19 pneumonia patients by damping dysregulated inflammation [11]. This tension, between the greater vulnerability to acquisition and the potential benefit during cytokine-driven lung injury, argues for individualized decisions on holding and restarting therapy, pausing tofacitinib during critical illness, and resuming after recovery, aligned with regulatory guidance and clinical judgment [10].

Reports with tofacitinib describe severe opportunistic pneumonias (*Pneumocystis jirovecii*, cytomegalovirus, and varicella-zoster), often with hypoxemic respiratory failure and ICU-level support, while the term "ARDS" is seldom explicitly used [23-26]. These observations align with the drug’s mechanism, blunting interferon-mediated antiviral defense via JAK-STAT inhibition, which can lower the threshold for viral lower-respiratory tract injury. To our knowledge, there is no prior published report of HRV-associated ARDS under tofacitinib; this case extends that spectrum.

Diagnostics, differential, and management in HRV-ARDS

Syndromic multiplex PCR was pivotal but required context. The BioFire panel reports a combined HRV/EV analyte from airway specimens. Positive results must be integrated with imaging and clinical evolution because colonization, prolonged shedding, or upper-lower tract discordance can occur [14]. In our patient, the HRCT pattern, monophasic course, and exclusion of alternatives supported causality.

Discriminating viral ARDS from MTX pneumonitis and RA-associated interstitial lung disease (RA-ILD) shaped management. Bedside decisions were anchored to the ICU-level discriminators compiled in Table 5. MTX pneumonitis often shows organizing pneumonia or non-specific interstitial pneumonia (NSIP) phenotypes and may relapse on re-challenge. Our patient’s viral prodrome, HRV/EV PCR positivity, and uneventful MTX re-initiation argued against drug toxicity [27]. RA-ILD typically evolves more indolently with usual interstitial pneumonia or NSIP patterns. The absence of pre-existing lung disease, the acute monophasic course, and full recovery without ILD-directed therapy made an RA-ILD flare unlikely [28,29].

**Table 5:** Practical differential of acute hypoxemic respiratory failure in RA in the ICU setting Practical ICU differential for an RA patient with acute hypoxaemic respiratory failure, contrasting viral ARDS, MTX pneumonitis, RA-ILD flare, and typical bacterial pneumonia across onset/trigger, HRCT hallmark, POCUS/LUS, early labs/biomarkers, first 48-72-hour treatment/response, and re-challenge/trajectory clues. “—” denotes not reported; “±” possible/present; and “→” indicates typical evolution [12,13,27-34]. ARDS: acute respiratory distress syndrome, BCx: blood cultures, GGO: ground-glass opacity, HFNC: high-flow nasal cannula, HRCT: high-resolution computed tomography, ICU: intensive care unit, ILD: interstitial lung disease, LUS: lung ultrasound, MTX: methotrexate, NIV: non-invasive ventilation, NSIP: nonspecific interstitial pneumonia, OP: organizing pneumonia, PCT: procalcitonin, PCR: polymerase chain reaction, POCUS: point-of-care ultrasound, RA: rheumatoid arthritis, RA-ILD: rheumatoid-arthritis-associated interstitial lung disease, UIP: usual interstitial pneumonia

ICU discriminator	Viral ARDS [12,13]	MTX pneumonitis [27]	RA-ILD flare [28,29]	Typical bacterial pneumonia [30]
Onset and trigger	Acute, viral prodrome	Subacute; weeks–months on MTX	Indolent/subacute; prior ILD risk	Acute; aspiration/community exposure
HRCT hallmark	Diffuse GGO ± dependent consolidation; mild septal lines [31]	GGO ± OP/NSIP pattern; peripheral	UIP/NSIP pattern; basal/peripheral fibrosis	Lobar/segmental consolidation, air bronchograms [32]
POCUS/LUS [33]	Diffuse B-lines; dependent consolidation	Diffuse B-lines; patchy subpleural	Irregular pleural line; focal B-lines	Focal consolidation with dynamic air bronchograms
Early labs/biomarkers	Viral PCR positive; low-intermediate PCT [34]	Eosinophilia may occur; infection workup negative	Autoimmune context; infection workup negative	Neutrophilia; higher PCT; positive sputum/BCx possible [34]
First 48–72 h treatment and response	HFNC/NIV, conservative fluids, prone; antibiotics de-escalate if cultures negative; short steroids sometimes used	Stop MTX; systemic steroids; exclude infection	ILD-directed care ± steroids; no pathogen	Empiric antibiotics → defervescence/radiographic improvement
Re-challenge/trajectory clue	Not applicable	MTX re-challenge → relapse	Chronic/recurrent course; fibrosis persists	Rapid response to antibiotics; relapse with new aspiration

Supportive ARDS care likely determined the outcome. HFNC and prolonged awake proning improved oxygenation and, in meta-trial data, lower intubation risk in selected acute hypoxemic respiratory failure [17,35,36]. If intubation becomes necessary, lung-protective ventilation, avoidance of injurious pressures, judicious positive end-expiratory pressure strategies, and early prone positioning remain foundational; current guidance also supports considering corticosteroids in established non-COVID ARDS [12,13,17,35-37]. Our short, early steroid course paralleled DEXA-ARDS evidence and coincided with steady improvement without superinfection [38].

No approved antiviral therapy exists for HRV. Investigational capsid binders (e.g., pleconaril) did not gain approval owing to safety and interaction concerns. Timely diagnosis and meticulous supportive care, therefore, remain the pillars of management [22,39,40].

Strengths and limitations

Strengths of this report include virologic confirmation with a syndromic multiplex PCR, close imaging-clinical concordance supporting viral ARDS rather than methotrexate pneumonitis or RA-ILD, and a transparent, day-by-day timeline linking diagnostics, respiratory support, antimicrobial stewardship, and outcomes. We also provide a focused mini-review of published adult HRV-ARDS, a structured differential with practical discriminators, and longitudinal follow-up demonstrating radiographic resolution after staged withdrawal and subsequent re-introduction of immunosuppression guided by shared decision-making.

Limitations are inherent to a single-patient observation: no lower-airway sampling or quantitative viral load/genotyping to definitively localize infection or distinguish HRV from EV on the panel; absence of bronchoscopy and lung histology; limited long-term physiologic follow-up (e.g., pulmonary function tests/diffusing capacity of the lung for carbon monoxide); and residual uncertainty about the independent contribution of corticosteroids versus the natural disease course. Although other pathogens were repeatedly unrevealed and the clinical trajectory favored HRV-ARDS, occult co-infections cannot be excluded with absolute certainty, limiting generalizability.

## Conclusions

Adult HRV-associated ARDS, though uncommon, should be considered in immunosuppressed patients with rapidly progressive hypoxemic respiratory failure. Rapid upper-airway multiplex PCR can detect the combined HRV/EV analyte, but results must be integrated with HRCT findings and the clinical trajectory. In patients receiving JAK inhibitors, pausing therapy during serious infection is prudent, with individualized resumption after recovery. Differentiation from methotrexate pneumonitis and RA-ILD hinges on timing, imaging phenotype, exclusion of alternative pathogens, and tolerance of methotrexate re-challenge.
